# Examining Effects of Age on Outcomes after Nontraditional Motorized Vehicle Accidents

**DOI:** 10.7759/cureus.9834

**Published:** 2020-08-18

**Authors:** Morghan Jameson, Christy Lawson, Hannah Wheeler, Matthew Leonard, Megan Quinn, Bracken Burns

**Affiliations:** 1 Medicine, East Tennessee State University Quillen College of Medicine, Johnson City, USA; 2 Surgery, East Tennessee State University Quillen College of Medicine, Johnson City, USA; 3 Trauma, Ballad Health Trauma Services, Johnson City, USA; 4 College of Public Health, East Tennessee State University, Johnson City, USA

**Keywords:** nontraditional motor vehicle, trauma, outcomes, elderly trauma

## Abstract

This study examined patient outcomes from accidents involving nontraditional motorized vehicles. A total of 558 patients aged >17 years were observed retrospectively. The study groups were divided by age. The Adult Trauma Group (ATG) (N=452) consisted of patients aged 18-64 years and the Elderly Trauma Group (ETG) (N=106) consisted of patients aged ≥65 years. All-terrain vehicle (ATV) accidents were the most common (N=437, 78%) among both study groups and also the primary cause of death, with 17 deaths (4%). The most common discharge disposition was home or self-care (routine discharge) with 427 patients (77%). The mortality rate of the total population was 4.1% (23 total deaths). There was a statistically significant difference in length of hospital stay (p=0.03) and length of Intensive Care Unit (ICU) stay (p=0.03) between the two groups and patients ≥65 years were statistically more likely to be discharged to a care facility vs. home. Nontraditional motorized vehicles continue to grow in popularity in all ages and the effect of age on patient outcomes after injury is an important area to evaluate.

## Introduction

Outdoor recreation and the use of nontraditional motorized vehicles have increased in popularity over the years. These types of vehicles have been used more commonly, specifically with farming, due to their ability to assist with a variety of agricultural-related tasks such as inspecting crops, plowing, and towing supplies. Based on observation at our Level 1 Trauma center, All-Terrain Vehicles (ATV), lawnmowers and tractors are among the most commonly used in rural areas due to farming and the availability of land for recreation. According to the Specialty Equipment Marketing Association (SEMA), the sale of Utility Task Vehicles (UTVs) has increased by 95.3% since 2006 [1]. These types of vehicles are used by people of all ages. Although safety components have improved over the years, injuries still tend to be a common cause of hospital trauma admissions. Lawnmower injuries continue to be a public health concern and it is estimated that 51,151 lawnmower accidents with emergency room (ER) visits occurred between 2006-2013 [2]. Tractor rollovers remain the number one cause of injury or death on a farm according to the Great Plains Center for Agricultural Health [3]. ATVs contributed to an estimated 81,800 accidents in all ages in 2018 according to the U.S. Consumer Product Safety Commission [4]. Less common accidents involving aircraft, watercraft, and industrial machinery also occur.

Due to the rapid growth of the elderly population over the past several decades, there has been an increased number of elderly patients requiring treatment for trauma injuries [5]. Age can have a significant impact on hospital outcomes and mortality in patients who are admitted to trauma [6]. According to the literature, the elderly population tends to develop more severe complications and are at greater risk of death compared to the younger population after traumatic injury [7]. This is likely contributed to preexisting comorbid conditions [7]. The elderly patient lacks the physiological reserve to respond to the stress of a traumatic injury and most organ systems have a decreased functional capacity, increasing the risk of shock [8]. At age 70, the mortality risk after a traumatic injury is three times that of a patient who is 20 years of age [9].

A search in the current literature reveals that there has been little research on the effect of patient’s age and health outcomes related to nontraditional motor vehicle accidents (NMVA). This study aimed to examine the age-related outcomes in patients who were involved in a NMVA. We hypothesize that the elderly patients involved in NMVA will demonstrate poor outcomes compared to the younger population.

## Materials and methods

This retrospective study was approved by the office for the protection of human research projects of East Tennessee State University. Electronic health data of trauma patients was obtained from the Johnson City Medical Center trauma department registry. Inclusion criteria are as follows: • >17 years of age • Primary reason for admission was injury due to nontraditional motorized vehicles (snowmobiles, lawnmowers, ATVs, machinery- industrial or agricultural, and any other nontraditional motorized vehicle that was not a car, truck or motorcycle). • Admission occurred between January 1st, 2011 and December 31st, 2019

The design of this study was an observational cross-sectional survey. The International Classification of Diseases, Ninth Edition (ICD-9) and Tenth Edition (ICD-10) were used to obtain parameters for the study. Age, hospital Length of stay (LOS), total length of stay in the intensive care unit (ICU), ventilator days, injury severity score (ISS), and mechanism of injury were obtained for each patient from the existent trauma registry. The patients were split into two study groups. The Adult Trauma Group (ATG) contained patients aged 18-64 and the Elderly Trauma Group (ETG) contained patients aged ≥65.

Descriptive statistics and regression analysis were used to compare the means of the study groups and p≤0.05 was considered statistically significant. A comparison between each study parameter (total length of stay, length of stay in the ICU, total vent days, and injury severity score) was done using t-test for differences in means in order to determine whether there was a significant difference between the specified study groups. Patient dispositions were combined into two separate categories (Disposition A: home/self-care and/or services, mental health facility, AMA Disposition B: acute care facility, inpatient rehabilitation, skilled nursing facility). Chi-square was done to test the relationship between the two groups and patient disposition.

## Results

**Figure 1:**
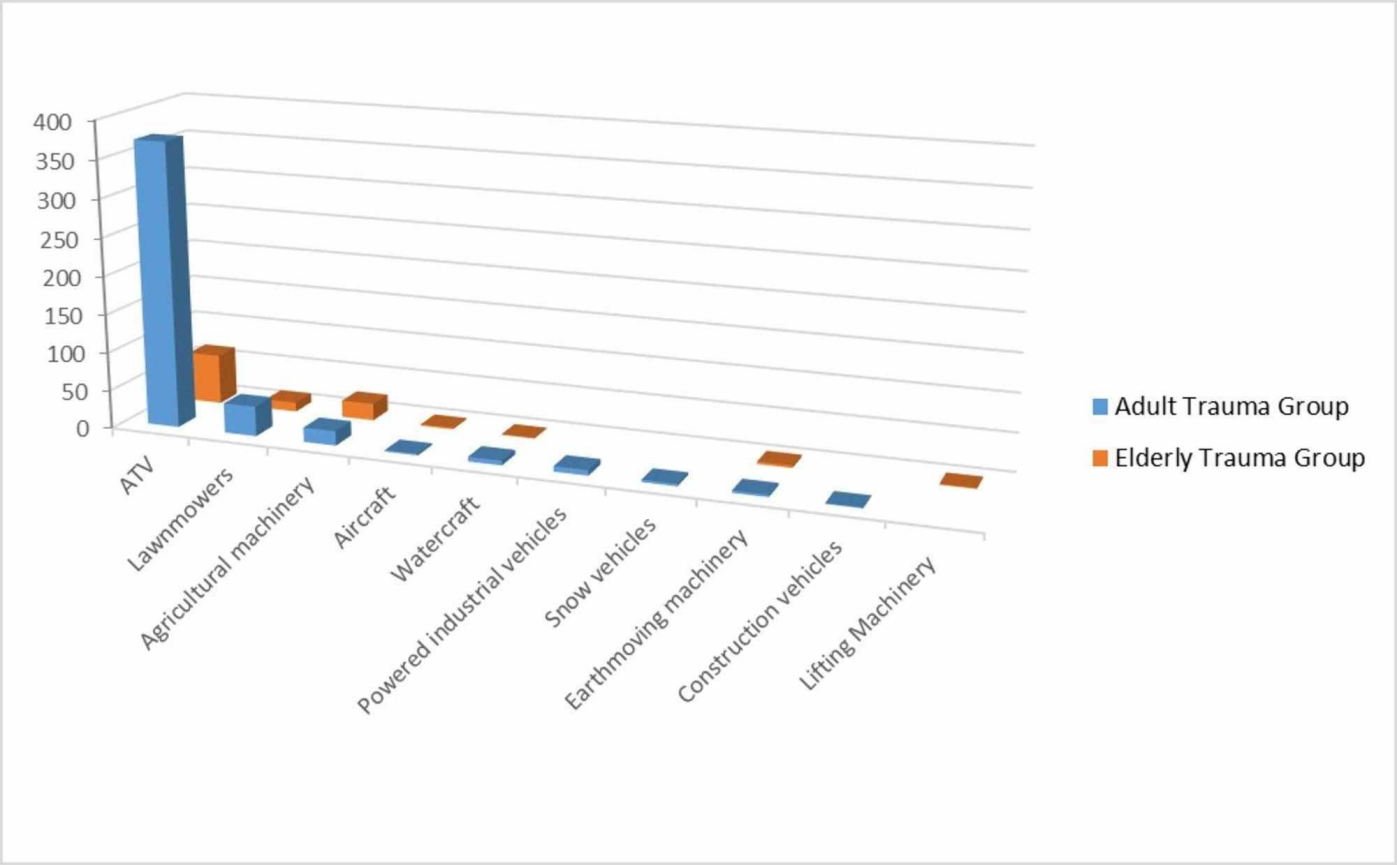
Mechanism of injury among patients

The ATG consisted of 452 patients between the ages of 18-64 years with a mean age of 39.7 years. The ETG consisted of 106 patients ≥65 years with a mean age of 75.0 years. The type of nontraditional motorized vehicle accidents varied from lawnmowers (ATG; N=39, ETG; N=12), agricultural machinery (ATG; N=19, ETG; N=22), aircraft (ATG; N=2, ETG; N=2), watercraft (ATG; N=6, ETG; N=1), lifting machinery (ATG; N=0, ETG; N=1) powered industrial vehicle (ATG; N=7, ETG; N=0), snow vehicles (ATG; N=3, ETG; N=0), earthmoving machinery (ATG; N=3, ETG; N=3) and construction vehicles (ATG; N=1, ETG; N=0) with the most common being ATV accidents (ATG; N=372 (82%), ETG; N=65 (61%)) (Figure 1).

**Figure 2:**
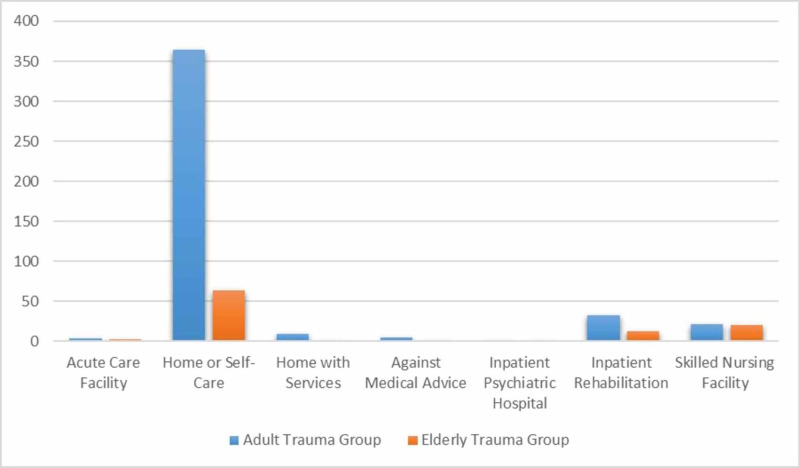
Patient disposition

Out of the 452 patients observed in the ATG, there were 437 (96.7%) survivors and 15 (3.3%) fatalities. Out of the 106 patients observed in the ETG, there were 98 (92%) survivors and eight (8%) fatalities. The most common cause of death in both groups was ATV accidents which made up 87% (13 deaths) of the total fatalities in the ATG and 50% (four deaths) of the total fatalities in the ETG. In both groups, home or self-care was the most common disposition for patients (ATG 83%, ETG 64%). Other dispositions include inpatient rehabilitation (ATG 8%, ETG 12%) and skilled nursing facility (ATG 5%, ETG 20%). Less frequent dispositions include acute care facility (ATG 1%, ETG 2%), against medical advice (ATG 1%, ETG 0%), and home with services (ATG 2%, ETG 1%). One patient in the ATG group was discharged to an inpatient psychiatric hospital. A breakdown of dispositions between the two groups is shown below (Figure 2).

There was a statistically significant difference in hospital length of stay (4.1 days vs 5.3 days; p=0.05) and mortality (ATG=3.4% vs ETG=6.0%; p=0.05) between the two groups. Mean ICU LOS (1.8 days vs 2.8 days; p=0.14) was greater in the ETG but there was no significant difference between the two groups. The mean number of ventilator days were similar and there was no significant difference (1.4 vs 1.6; p=0.79). The maximum ISS was lower in the ATG compared to the ETG (45 vs 75) but the mean ISS score was similar in both groups and there was no significant difference (9.9 vs 11.6; p=0.12) (Table 1). A chi-square test demonstrated there was a statistically significant difference (p<0.001) between the two groups demonstrating that patients ≥65 years of age more likely to be discharged to an acute care facility, inpatient rehabilitation, or skilled nursing facility compared to the patients 18-64 (Table 2).

**Table 1:** Comparison of ATG and ETG ATG: Adult Trauma Group, ETG: Elderly Trauma Group, ISS: injury severity score

	ATG		ETG		
	Frequency	Percent	Frequency	Percent	P value (≤0.05)
Sample size	452		106		
Longest hospital stay (days)	46		37		
Mean hospital stay (days)	4.1		5.3		0.05
Longest ICU stay (days)	32		22		
Mean ICU stay (days)	1.8		2.8		0.14
Longest Ventilation days	32		19		
Mean ventilation days	1.4		1.6		0.79
ISS	0-45		0-75		
Mean ISS	9.9		11.6		0.12
Mortality	14	3.4%	9	6.0%	0.05

**Table 2:** Comparison of disposition of the ATG and the ETG Disposition A: home/self-care and/or services, mental health facility, AMA Disposition B: acute care facility, inpatient rehabilitation, skilled nursing facility ATG: Adult Trauma Group, ETG: Elderly Trauma Group

	ATG	ETG	
			P value
Disposition A	379	64	
Disposition B	58	34	
			P<0.001

## Discussion

As the use of non-traditional motor vehicles increase in popularity, the increased incidence of trauma cases of all ages are following this pattern. Age is a well-known risk factor in trauma, and over the past few decades the average age of the trauma patient is increasing. We have specifically seen an increase in elderly trauma related to NMVA at our Level 1 Trauma Center. Our aim was to investigate whether or not age could have an impact on the outcome of trauma patients admitted after a NMVA. Our study suggests elderly patients were found to have longer length of stay and increased mortality. Elderly patients were found to have longer length of hospital stay and higher mortality. Patient disposition is also strongly correlated with age with elderly patients more likely to be discharged to skilled nursing/rehab facility. No statistical significance were found between the ATG and the ETG in correlation to ICU stay, ISS, or ventilator days. Being a single institution retrospective study we recognize our findings may not be able to be generalized to the entire population.

There are over 9.2 million ATVs in operation across the United States today, which would explain why our findings demonstrated the most common NMVA and cause of death in both age groups were accidents involving ATVs [10]. In a review of the literature, a majority of off-road vehicle accident research is focused on the adolescent population and very few studies examine outcomes in age >18 years and the older population. One study done by Deladisma et al. compared outcomes of ATV accidents in geriatrics and their younger counterparts. Similar to our results, they found that the geriatric population had a significantly longer hospital length of stay and age over 60 years was an independent predictor of mortality [11]. They also found that the geriatric population had a longer ICU length of stay, which differed from our results [11]. A study done by Adams et al. demonstrated that geriatric ATV riders involved in accidents trended towards longer hospital stays and higher mortality (12.5% v 3.45%) compared to the younger population, however this was not significant [12]. Another study completed by Taylor et al. demonstrated that patients ≥65 years of age who had experienced blunt trauma had significantly longer hospital stays and higher mortality rates than younger trauma patients [13]. On the contrary, another study examining patients admitted for orthopedic trauma demonstrated no significant difference in hospital length of stay in the younger age group compared to patients 70 years and older; however, those patients 70 years and older had a significantly longer ICU stay than the younger age group [14]. 

According to the literature, age could be a strong predictor in a patient’s ability to return home and perform activities of daily living (ADL) independently post-discharge. Our results demonstrate that patients who are ≥65 years are more likely to be discharged to a care facility compared to the younger population. This could represent an ongoing impact on personal and system resources, increasing the cost of care. A recent study done by Goldway et al. demonstrated results similar to ours showing that patients ≥65 years were more likely to be discharged to a facility after an ATV or snowmobile accident compared to younger adults [15]. Another literature review study also demonstrating similar results showed that older trauma patients had more comorbidities, higher ISS, longer length of hospital stay, and more pain than their younger counterparts [16]. Those patients who experienced more pain and longer hospital stay were more likely to be discharged to a facility [16]. On the other hand, a study looking at patients demonstrated after serious injury from blunt trauma, 89% of the older adult patients were discharged home with minimal assistance with ADLs. However, those patients who returned home were younger, had less severity of injury, had shorter hospital stays, and fewer complications [17]. Another study explored patients over the age of 65 years using frailty scores to predict discharge disposition. They determined that patients who were frailer were more likely to be discharged to a facility vs home after trauma. Frailty could be a beneficial tool in predicting outcomes and disposition post-trauma in patients of all ages [18]. 

Based on what we have seen at our Level 1 Trauma center in regards to an increase in NMVA in the elderly population we felt it would be beneficial to focus on this age group. Data for the elderly population and NMVA is limited in the literature. Our rural community has an increasing population of aging farmers and laborers who have traditionally utilized nontraditional motor vehicles as part of their work routine. Knowing that elderly patients in our study are more prone to longer hospital stays, higher mortality, and higher likelihood of discharge to a facility, we can focus on injury prevention efforts in the future. Knowing this can also help with disposition planning if you already know they are likely to be discharged to a skilled nursing facility or rehab - getting case management involved earlier with planning can decrease hospital LOS.

## Conclusions

In this study the ETG was more likely to have a longer hospital stay and higher mortality rate compared to the ATG. Also, the ETG was more likely to be discharged to a care facility. Our future efforts will be focused on awareness and injury prevention in this age group.
